# Three new species and a new genus of majoid crabs from the eastern Pacific (Decapoda, Brachyura)

**DOI:** 10.3897/zookeys.825.32271

**Published:** 2019-02-18

**Authors:** Jessica Colavite, Amanda Windsor, William Santana

**Affiliations:** 1 Universidade do Sagrado Coração, Pró-Reitoria de Pesquisa e Pós-Graduação, Laboratório de Sistemática Zoológica, 10-50, Irmã Arminda st., Jd. Brasil, Bauru, SP 17011-160, Brazil Universidade do Sagrado Coração Bauru Brazil; 2 Departamento de Zoologia, Instituto de Biociências, Universidade do Estado de São Paulo (UNESP), Botucatu, SP 18618-970, Brazil Universidade do Estado de São Paulo Botucatu Brazil; 3 Department of Invertebrate Zoology, National Museum of Natural History, Smithsonian Institution, Museum Support Center, 4210 Silver Hill Road, Suitland, MD 20746-2863, USA National Museum of Natural History, Smithsonian Institution Suitland United States of America

**Keywords:** Biodiversity, Epialtidae, Inachoididae, Pisinae, spider crab

## Abstract

Three new species and a new genus of majoid crabs from deep waters in the eastern Pacific are described and illustrated using morphological and molecular data. A new species of inachoidid, *Collodesanartius***sp. n.** is described from Peru, which resembles *C.tenuirostris* Rathbun, 1893, in the general appearance of the carapace, but is distinguished by the details of tubercles on the carapace and thoracic sternum, proportions of the pereopod articles, and bathymetric distribution. A new epialtid, *Nibiliamachala***sp. n.**, is described from Ecuador; *Nibilia* A Milne-Edwards, 1878 has, until now, been considered to be monotypic, occurring only in the western Atlantic. This new species, from the eastern Pacific, closely resembles *N.antilocapra* (Stimpson, 1871) in the general morphology, but can be distinguished by the number of spines on the carapace and pereopods. Another epialtid, *Solincaaulix***gen. n. et sp. n**, is establish for material collected from Ecuador and Peru, and can be easily identified from other taxa by the presence of a deep furrow between the very inflated branchial regions.

## Introduction

The Southeast Pacific Biological Oceanographic Project (SEPBOP) comprised several cruises of the R/V Anton Bruun, between October 1965 and September 1966 (Child 1992; Garth and Haig 1971). Examination of unidentified majoids from this expedition in the National Museum of Natural History, Smithsonian Institution (USNM) Crustacea Collection revealed the existence of three new species and a new genus of spider crabs from southeast Pacific Ocean. Material from this cruise were found to be viable for DNA sequencing so, in addition to morphological analyses, we constructed a multi-locus molecular phylogeny of these three new taxa to place them within the context of other members of their respective genera and within the superfamily Majoidea.

The amphi-American inachoididae *Collodes* Stimpson, 1860, currently comprises 15 species, four of which are known from the eastern Pacific, inhabiting waters up to 700 m deep (Santana and Tavares 2017). The new species, *Collodesanartius* sp. n., is described from the northwest of Peru, based on 118 specimens.

The second new species described herein belongs to the epialtid *Nibilia* A Milne-Edwards, 1878. *Nibiliamachala* sp. n. is described from a single female collected in waters off Machala, Ecuador. Until now, *Nibiliaantilocapra* (Stimpson, 1871) was considered to be the only species of the genus, which is found in waters between 71–342 m on muddy and sandy bottoms with broken shells, corals, and rocks in the western Atlantic (Melo 1996, Hernández-Ávila et al. 2008, Carmona-Suárez and Poupin 2016).

A new genus, *Solinca* gen. n. established for *Solincaaulix* gen. n. et sp. n. is based on morphological analysis of 28 specimens from three different localities between Ecuador and Peru. *Solincaaulix* gen. n. et sp. n. is phylogenetically allied to Epialtidae crabs *Scyraacutifrons* Dana, 1851, *Pugettianipponensis* Rathbun, 1932, *Pugettiaquadridens* (De Haan, 1839) and *Chorilialongipes* Dana, 1851. *Solincaaulix* gen. n. et sp. n. shares the distinct, sculpted chelipeds with these allied species. *Solincaaulix* gen. n. et sp. n., nevertheless, can be easily distinguished by a unique set of characters.

## Materials and methods

Holotype and paratype specimens were deposited in the National Museum of Natural History, Smithsonian Institution, Washington, DC, USA (**USNM**). Additional paratype specimens of *Collodesanartius* sp. n. and *Solincaaulix* gen. n. et sp. n. are deposited in the Museum of Zoology at the University of São Paulo (**MZUSP**). Comparative material for both morphological and molecular assessments was obtained from the University of Louisiana at Lafayette Zoological Collection (**ULLZ**), Museu de Oceanografia Prof. Petrônio Alves Coelho (**MOUFPE**), MZUSP, and USNM. The terminology used follows Davie et al. (2015). Abbreviations used:

**cl** carapace length, taken along the dorsal midline from the base of the rostral sinus to the posterior margin of the carapace;

**cw** carapace maximum width, taken at the level of its widest point, branchiostegal spines excluded;

**G1** first gonopod or male pleopod 1;

**G2** second gonopod or male pleopod 2;

**P2–P5** pereopods 2 to 5 (P1 is the cheliped).

**R/V** research vessel.

### DNA extraction, PCR, and sequencing

Total genomic DNA was extracted from muscle tissue using either the Qiagen DNeasy Blood and Tissue extraction kit or an Omega Bio-tek EZNA Tissue DNA Kit. Partial sequences of the 12S, 16S, and barcode region of COI mitochondrial genes were amplified with the following primers respectively: 12SF (Mokady et al. 1994) and 12S1R (Shull et al. 2005), 16SF/16SR (Hultgren and Stachowicz 2008), and LCO1490/HCO2198 (Folmer et al. 1994). The nuclear loci histone-H3 (H3AF/H3AR, Colgan et al. 1998) and the small subunit 18S rRNA (A/L, C/Y, O/B of Medlin et al. 1988, Apakupakul et al. 1999, or B/D18s1R, D18s2FD18s2R, D18s3F-D18s3R, D18s4F-D18s4R and D18s5F-A of Bracken et al. 2009) were also sequenced from supporting majoid taxa. Annealing temperatures for PCRs were 58 °C, 54 °C, and 48 °C for 12S/18S, 16S/H3, and COI, respectively. Reagent volumes and concentrations followed manufacturer instructions; primer concentrations were 10 µM. Sequencing reactions were performed using 1 µL of purified PCR product in a 10 µL reaction containing 0.5 µL primer, 1.75 µL Big Dye buffer and 0.5 µL BigDye (Life Technologies). Reactions were purified using Millipore Sephadex plates (MAHVN–4550) according to the manufacturer’s instructions and sequenced on the ABI 3730XL automated DNA sequencer. Sequences were assembled, trimmed of primers, and checked for quality using Geneious 9.1.8.

### Molecular data analysis

Sequences generated for this study were combined with those from Windsor and Felder (2014, 2017), other sequences available from GenBank, and previously unpublished sequences generated by A Windsor in order to place the new taxa, particularly *Solincaaulix* gen. n. et sp. n, within the context of Majoidea. Locality information and GenBank accession numbers for taxa included in the molecular analyses are provided in Suppl. material 1: Table S1.

**Figure 1.**
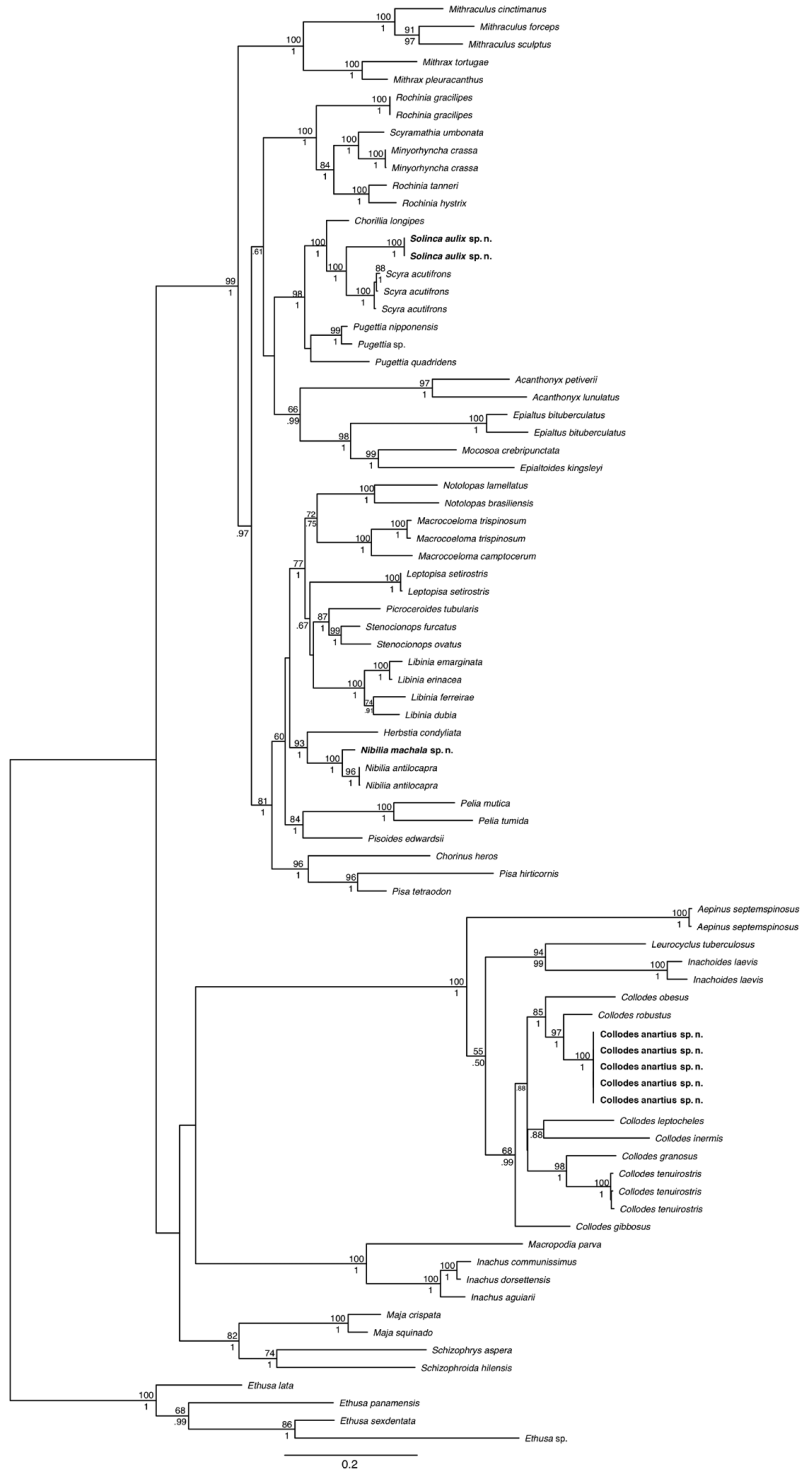
Molecular phylogenetic tree represented as maximum likelihood topology of three mitochondrial and two nuclear genes (12S, 16S, COI, 18S, H3) to place *Collodesanartius* sp. n., *Nibiliamachala* sp. n. and *Solincaaulix* gen. n. et sp. n. within the context of Majoidea based on 32 genera. Node support values are shown with maximum likelihood bootstrap support above line and Bayesian posterior probabilities below line.

Multiple sequence alignment was performed using the MAFFT FFT-NS-I (Katoh et al. 2005) alignment algorithm for the individual molecular markers. The individual datasets were concatenated in SequenceMatrix (Vaidya et al. 2011) and the perl script PartitionFinder (Lanfear et al. 2017) was run to determine the appropriate model of evolution and partitioning scheme. Phylogenetic trees were constructed using maximum likelihood in RAxML 7.0.4 (Stamatakis 2006) and Bayesian inference (BI) in MrBayes (v3.2.1) (Huelsenbeck and Ronquist 2001). In RAxML, we used the ‘-f ae’ option with 1000 bootstrap replicates. Likelihood parameters followed the General Time Reversible (GTR) model with a gamma distribution on the partitioned dataset and RAxML estimated all free parameters. The resulting best tree was used to reflect phylogeny (Fig. 1). Bayesian inference was performed with 10,000,000 generations with a 25% burn-in and sampling every 1000 generations. A mixed model was applied to the partitioned dataset. A 50% majority-rule consensus tree was constructed from the post-burn-in trees. DNA extraction and sequencing for *Collodesanartius* sp. n., *Nibiliamachala* sp. n. and *Solincaaulix* gen. n. et sp. n. was carried out at the Smithsonian Institution’s Laboratories of Analytical Biology, and phylogenetic trees were generated on the Smithsonian Institution High Performance Computing Cluster (SI/HPC).

## Results

All three mitochondrial loci (12S, 16S, COI) were successfully amplified and sequenced for the three new species. Nodes where maximum likelihood bootstrap support and Bayesian posterior probabilities greater than 50/0.5 are shown on the maximum likelihood phylogram (Fig. 1). *Nibiliamachala* is supported (100/1) as sister to *N.antilocapra* and *Nibilia* is sister to *Herbstiacondyliata* (Fabricius, 1787) with high support (93/1). However, their position within a clade comprised of Atlantic members of Pisinae sensu Ng et al. (2008) is unresolved. *Solincaaulix* is highly supported (100/1) as sister to *Scyraacutifrons* within a well-supported subclade containing *Pugettia* spp. and *Chorilia*. Both *Nibilia* and *Solinca* are nested within Epialtidae sensu Ng et al. (2008). *Collodesanartius* is represented here by five individuals that are highly supported (100/1) as sister to *C.robustus* (97/1). Interestingly, *C.anartius* is more closely allied to Gulf of Mexico/western Atlantic Ocean species than to *C.tenuirostris*, which it closely resembles.

## Systematics

### Superfamily Majoidea Samouelle, 1819

#### Family Inachoididae Dana, 1851

##### Collodes

Taxon classificationAnimaliaDecapodaInachoididae

Stimpson, 1860

Collodes Stimpson, 1860: 193, pl. II, fig. 4. [Type species: Collodesgranosus Stimpson, 1860, by original designation and monotypy].

###### Included species.

*Collodesanartius* sp. n.; *Collodesarmatus* Rathbun, 1898; *C.gibbosus* (Bell, 1835) (formerly in *Mycrorhynchus*); *C.granosus* Stimpson, 1860; *C.inermis* A Milne-Edwards, 1878; *C.leptocheles* Rathbun, 1894; *C.levis* Rathbun, 1901; *C.nudus* Stimpson, 1871; *C.obesus* A Milne-Edwards, 1878; *C.robsonae* Garth, 1958; *C.robustus* Smith, 1883; *C.rostratus* A Milne-Edwards, 1878; *C.tenuirostris* Rathbun, 1893; *C.trispinosus* Stimpson, 1871 (= *Collodesdepressus* A Milne-Edwards, 1878 junior subjective synonym); *C.tumidus* Rathbun, 1898; *C.tuerkayi* Santana & Tavares, 2017.

##### Collodes anartius sp. n.

Taxon classificationAnimaliaDecapodaInachoididae

http://zoobank.org/DE0C558B-A35A-4E21-903F-35B83C026485

**Figure 2.**
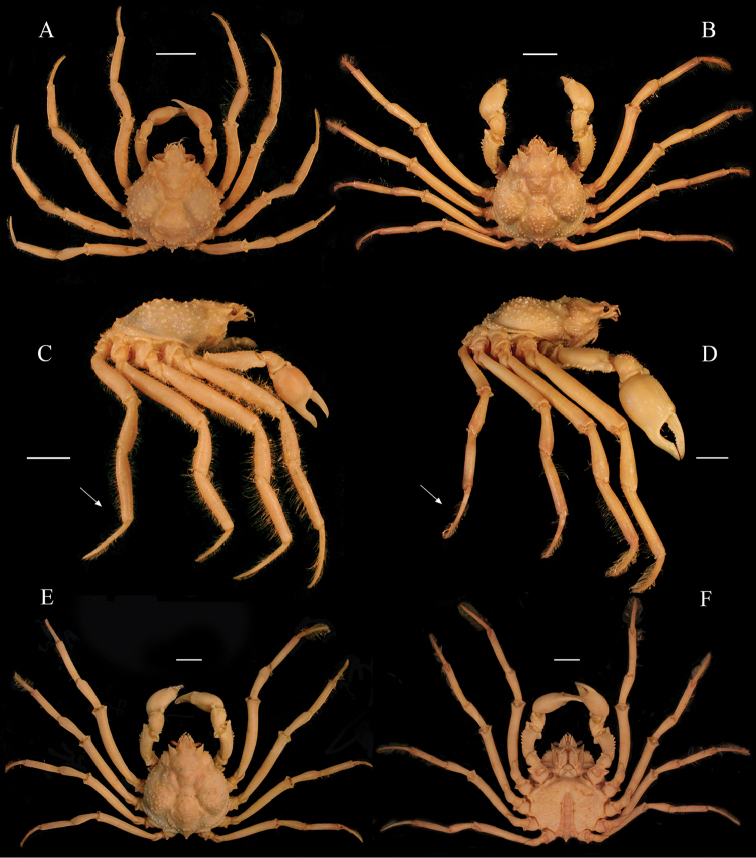
*Collodesanartius* sp. n., male paratype, cl 28.4 mm, cw 24.0 mm (USNM 142821) (**A, C**); male holotype, cl 27.0 mm, cw 23.5 mm (USNM 1462817) (**E, F**). *Collodestenuirostris* Rathbun, 1893, male, cl 36.2 mm, cw 29.5 mm (USNM 1462819) (**B, D**). Habitus, dorsal view (**A, B, E**). Lateral view (**C, D**). Ventral view (**F**). Note the proportions of the dactylus and propodus of P5 in both species (white arrows) (**C, D**). Scale bars: 10 mm.

**Figure 3.**
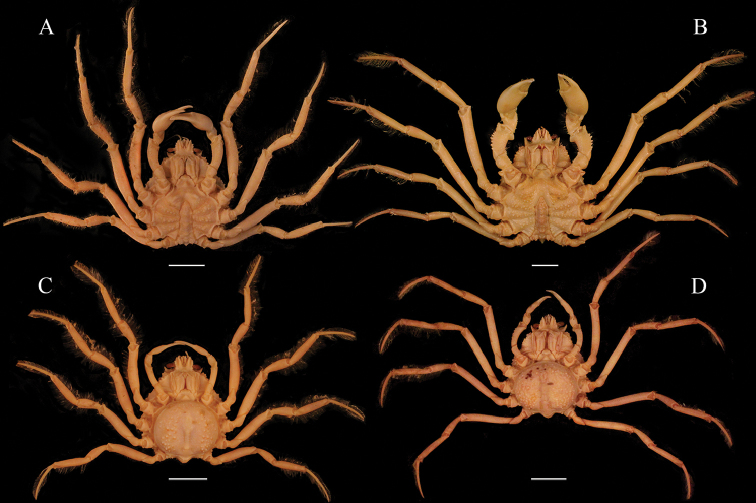
*Collodesanartius* sp. n., male paratype, cl 28.4 mm, cw 24.0 mm (USNM 142821) (**A**); Female paratype, cl 24.0 mm, cw 19.2 mm (USNM 142821) (**C**). *Collodestenuirostris* Rathbun, 1893, male, cl 36.2 mm, cw 29.5 mm (USNM 1462819) (**B**); Female, cl 25.25 mm, cw 20.51 mm (USNM 1462819) (**D**). Ventral view (**A–D**). Scale bars: 10 mm.

**Figure 4.**
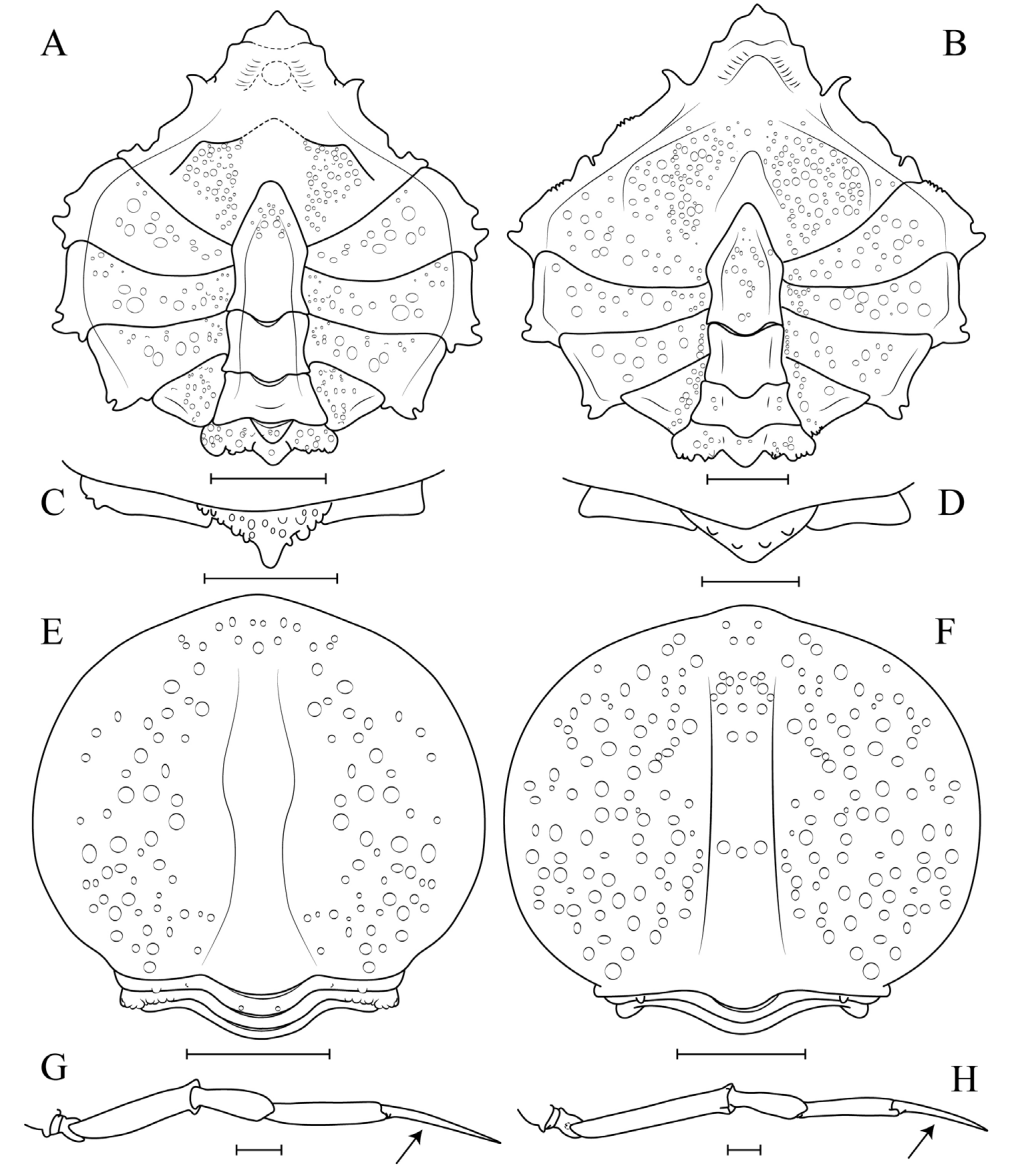
*Collodesanartius* sp. n., male paratype, cl 28.4, cw 24.0 (USNM 1462821) (**A, C, G**); Female paratype, cl 24.0 mm, cw 19.2 mm (USNM 1462821) (**E**). *Collodestenuirostris* Rathbun, 1893, male, cl 36.2 mm, cw 29.5 mm (USNM 1462819) (**B, D, H**); Female, cl 25.2 mm, cw 20.5 mm (USNM 1462819) (**F**). Cephalothorax ventral view (**A, B, E, F**); Ventral view of the first pleonal somite (**C, D**); Lateral view of the pereiopod 5 (P5) (**G, H**). Note the proportions of the dactylus of P5: longer than the propodus in *Collodesanartius* sp. n. (black arrow) (**G, H**). Scale bar: 5 mm.

Figures 2A, C, E, F ; 3A, C ; 4A, C, E , 7A, B

###### Holotype.

Peru, off Paita, Piura, Southeast Pacific Biological Oceanographic Project (SEPBOP), R/V Anton Bruun, cruise 16, stn 625–A, 04°57' / 05°01'S; 81°23'W, 02.vi.1966, Smithsonian Oceanographic Sorting Center coll., 118–133 m, male, cl 27 mm, cw 23.5 mm (USNM 1462817).

###### Paratypes.

Peru, off Paita, Piura Southeast Pacific Biological Oceanographic Project (SEPBOP), R/V Anton Bruun, cruise 16, stn 625–A, 04°57' / 05°01'S; 81°23'W, 02.vi.1966, Smithsonian Oceanographic Sorting Center coll., 118–133 m, 49 males, 7 females (USNM 1462747). Idem, 1 male (MZUSP 38889) 1 female (MZUSP 38890). Southeast Pacific Biological Oceanographic Project (SEPBOP), R/V Anton Bruun, cruise 16, stn 626–B, 05°07' / 04°59'S; 81°27'W, 03.vi.1966, Smithsonian Oceanographic Sorting Center coll., 365–457 m, male, cl 28.47 mm, cw 24.0 mm, 1 female, cl 24.0 mm, cw 19.2 mm, illustrated (USNM 1462821). Idem, 16 males, 2 juvenile females, 2 females, 32 ovigerous females (USNM 1462818). Southeast Pacific Biological Oceanographic Project (SEPBOP), R/V Anton Bruun, cruise 6, stn 627–A, 05°01'/ 04°59'S; 81°25'W/ 81°25'W, 03.vi.1966, Smithsonian Oceanographic Sorting Center coll., 200–311 m, 4 males, 1 female (USNM 1462676).

###### Comparative material.

*Collodestenuirostris*. Mexico, Sonora, Puerto Lobos, R/V Albatross, stn 3018, 30°16'00"N; 133°05'00"W, 24.iii.1889, USFC coll., MJ Rathbun det., 66 m, male holotype, 1 male paratype (USNM 17333). Costa Rica, off Playa Flamingo, R/V Urraca, stn CR–23, 15.vii.2005, R Collin coll., 1 male, DNA only (ULLZ 8235). Gulf of Nicoya, vii.1979, 9°48.27'N; 85°08.43'W, 25 m, 2 ovigerous females (USNM 1462759). Panama, Gulf of Panama, R/V Shimada, stn 63, 08° 11'N; 79°36'W, 13.iv.1967, A Windsor det., 4 ovigerous female (USNM 1479280). Ecuador, Manabi, off Cape San Lorenzo, Southeast Pacific Biological Oceanographic Project (SEPBOP), R/V Anton Bruun, cruise 18B, stn 775, 01°05'S; 80°98'W, 12.ix.1966, Smithsonian Oceanographic Sorting Center coll., 185 m, 1 juvenile male (USNM 1155058). Peru, west of Paita, Piura, Southeast Pacific Biological Oceanographic Project (SEPBOP), R/V Anton Bruun, cruise 16, stn 624–E, 04°51' / 04°57'S; 81°20' / 81°23'W, 02.vi.1966, Smithsonian Oceanographic Sorting Center coll., 79–91 m, 4 males, 5 ovigerous female, 1 juvenile female (USNM 1462819). West of Paita, Piura, Southeast Pacific Biological Oceanographic Project (SEPBOP), R/V Anton Bruun, cruise 16, stn 625–A, 04°57' / 05°01'S; 81°23'W, 02.vi.1966, 118–133 m, 1 ovigerous female, DNA only (USNM 1479343).

*Collodesgibbosus*. Costa Rica, off Playa Flamingo, R/V Urraca, stn CR–18, 15.vii.2005, R Collin coll., 15.5 m, 1 male, DNA only (ULLZ 8229). Ecuador, Salango Bay, Salango Island, R/V Velero III, Allan Hancock Pacific Expedition, stn 396–35, 18.i.1935, J Garth det., 22 m., neotype, female (USNM 100921).

*Collodesgranosus*. Mexico, Baja California, San Lucas Bay, Cabo San Lucas, R/V Albatross, stn 5681, 23.iii.1911, MJ Rathbun det., 24 m, 1 ovigerous female (USNM 55766). Panama, Panama Bay, R/V Urraca, 24.ii.2007, DL Felder coll., 20–30 m, 1 male, DNA only (ULLZ 9760).

*Collodesrobsonae*. Costa Rica, Gulf of Nicoya, 16.ii.1980, Dean & Howe coll., 70 m, 1 ovigerous female (USNM 1462812).

*Collodestumidus*. Mexico, Baja California, Magdalena Bay, R/V Albatross, stn 2831, 24°32'00"N; 111°59'00"W, 02.v.1888, MJ Rathbun det., 22 m, holotype, male (USNM 21571).

###### Type-locality.

Peru, west of Paita, Piura, 04°57'S to 05°07'S; 81°23'W to 81°27'W, 118–133 m.

###### Diagnosis.

Carapace pyriform, granulose, particularly on cardiac, branchial and intestinal regions. Second antennal article ventrolateral surface with strong, unarmed longitudinal keel. Third maxillipeds granulated; ischium and carpus with dense granulation, propodus smooth. First pleonal segment with a short spine or distinctly strong tubercle with several small tubercles around. P2–P5 dactylus smooth ventrally; dactylus of P5 longer than propodus; carpus of P5 more than half of merus.

###### Description.

Carapace pyriform, longer than wide; dorsal surface covered by tubercles of different sizes (more prominent in females), particularly on cardiac, branchial and intestinal regions; gastric and branchial regions covered with hooked setae. Gastric, branchial, cardiac, intestinal regions with small tubercles. Gastric, hepatic, branchial, cardiac, intestinal regions clearly delimited laterally by grooves. Metagastric region with mesial prominent tubercle; gastric region with few small tubercles; mesocardiac region with four tubercles in line longitudinally, distal tubercles more prominent. Groove between cardiac and intestinal region smooth or with a few small tubercles in males and females. Hepatic, branchial, intestinal regions densely covered with sub-equal tubercles. Metabranchial region slightly depressed. Thoracic pleurites V–VIII gymnopleura not fused to one another, usually densely covered with small hooked setae.

Rostrum simple, short, slightly curved upwards. Supraorbital spine absent; orbital margin unarmed dorsally. Postorbital spine longer than the ocular peduncle, directed laterally and upwards around eyes. Antennular fossae longitudinally ovate; anteroventral margin unarmed. Interantennular septum longer than epistomial spine, compressed laterally, forming ventrally-directed keel. Antenna (flagellum included) distinctly exceeding rostral length. First and second antennal articles fused to epistome. Second article protruding anterolaterally; ventrolateral surface with strong longitudinal keel, unarmed; ventrolateral margin dentate, teeth acute. Third antennal article massive; fourth longest, cylindrical; fifth article smaller.

Epistome markedly wider than long. Epistomial spine separated by small gap from interantennular septum. Mouthfield sub-rectangular. Pterygostomial region subtriangular, smooth near mouth frame, densely tuberculated laterally, tubercles sub-equal. Sub-hepatic region strongly swollen, delimited from pterygostome by distinct slope, densely covered with sub-equal tubercles, several long setae.

Third maxillipeds almost completely covering buccal frame, ischia leaving distinct gap. Exopod long, nearly reaching distal margin of merus; granulated; lateral margin with strong lobe in proximal third. Ischium distinctly longer than broad, dorsal face with longitudinal, smooth, deep groove; with granules; mesial margin slightly convex; crista dentata with small, rounded, irregularly sized teeth. Merus slightly longer than half of ischium, granulated. Anterior margin deeply incised, anterolateral and anteromedial margins expanded, mesial margin with a row of setae. Palp cylindrical, slightly overreaching ischiomeral suture. Carpus granulated; propodus and dactylus smooth, fringed with row of long setae.

Thoracic sternites II–IV broadly triangular in males, with sparse hooked setae, small tubercles medially. Anterior half of sternites I–IV strongly sloping down laterally, forming prominent triangle medially; fourth sternite densely tuberculated, with smaller projection ventrally directed in males. Male sternites IV–VII covered with distinct, large tubercles, outside sterno-pleonal cavity; smooth in females.

Male and female chelipeds sub-cylindrical, homochelous, robust in males, slender in females. Dactylus (movable) and fixed finger approximately same length as palm in males and females, covered with sparse setae. Males with finger cutting edges with small teeth, sub-equal in distal half, leaving distinct proximal depressed gap in sub-proximal edge of dactylus. Distal two-thirds of finger cutting edges with sub-equal teeth in females. Male propodus conspicuously inflated, with sparse setae, small tubercles in dorsal margin, fewer tubercles in ventral margin. Propodus slender, smooth in females. Carpus setose, with several tubercles dorsally, unarmed in females. Merus with two rows of small tubercles in dorsal and mesoventral faces, row of strong tubercles in lateral face, with long setae. Ischium sparsely tuberculate, setose. P2–5 similar in shape, slender, cylindrical; P2 longest, P3–P5 progressively decreasing in length. Dactylus of P3–P5 longer than propodus, without tubercles, carpus more than half of merus. Dactylus, propodus, carpus, merus densely setose, covered with long setae interspersed with hooked setae.

Male pleonal somites I–V free, sixth fused to telson. Pleotelson sub-triangular, rounded distally. Female pleonal somites I–IV free, somites V, VI, telson fused; pleotelson markedly arched, transversally oval. Male and female first pleonal somite with short spine or strong tubercle with several tubercles surrounding. Male somite I densely tubercular; somite VI-telson with scattered tubercles in the anterior margin. Female somites II–IV tubercular laterally; somites V–VI, telson evenly, densely tubercular in mesolateral region.

###### Distribution.

Northwest of Peru, from 04°57'S to 05°01'S; 81°23'W, 118 to 457 m.

###### Etymology.

The specific epithet is derived from the Greek adjective *anartius* for “uneven”, alluding to the rough similarity between the new species and *C.tenuirostris* and contours of the carapace.

###### Remarks.

Although the phylogenetic analyses of seven of the 15 described species of *Collodes* pointed to *C.robustus*, a western Atlantic species, as the sister species of *C.anartius* they are very distinct morphologically, with characters that clearly differentiate both species. For instance, (i) dorsal surface covered by tubercles of different sizes (more prominent on females) in *C.anartius* (vs. carapace evenly covered by small, similar in size tubercles in males and females of *C.robustus*); (ii) sternites V–VII with few, distinct, large tubercles in *C.anartius* (vs. sternites IV–VII with numerous, small, evenly distributed tubercles in *C.robustus*).

*Collodesanartius* superficially resembles *C.tenuirostris* Rathbun, 1893, in the general morphology with respect to size and distribution of carapace tubercles, the postorbital spines are also very similar in both species (Fig. 2A, B). Because they inhabit the same substrates, mud bottoms, and have the habit of completely camouflaging in the sediment, these species are difficult to separate. The bathymetric range was nevertheless different for both species in the Peruvian coast, wherein *C.anartius* can be found in deeper regions (118–457 m) than *C.tenuirostris* (25–133 m) (vide material examined).

Morphological characters that can distinguish *C.anartius* from *C.tenuirostris*, are: (i) third maxillipeds granulated; ischium and carpus with dense granulation, propodus smooth (in *C.anartius*) (vs. third maxilliped ischium and carpus sparsely granulate; propods granulate in *C.tenuirostris*) (Fig. 2A, B); (ii) first pleonal segment with a short spine or strong tubercle with several small tubercles around in *C.anartius* (vs. pleonal spine usually longer, with very few small tubercles around it in *C.tenuirostris*) (Fig. 2C, D); (iii) in males of *C.anartius* the fourth sternite has a small projection ventrally directed (vs. inflated projection in the fourth sternite in *C.tenuirostris*) (Figs 3A, B; 4A–D); (iv) all segments of female pleon of *C.anartius* are narrower, with fewer tubercles than in *C.tenuirostris* (Fig. 4E, F); (v) cutting edges of male cheliped fingers with small, sub-equal teeth in distal half and a distinct depressed gap distally in *C.anartius* (vs. well defined teeth in the cutting edges of fingers up to the distal gap in *C.tenuirostris*) (Fig. 2A, B); (vi) P2–P5 dactylus smooth ventrally in *C.anartius* (vs. P2–P5 dactylus with very small spines ventrally in *C.tenuirostris*, sometimes the spines are worn) (Fig. 2C, D); (vii) dactylus of P5 longer than propodus (vs. P5 dactylus and propodus of same size or dactylus smaller than propodus in *C.tenuirostris*) (Figs 2C, D; 4G, H); (xi) carpus of P5 more than half of merus in *C.anartius* (vs. carpus of half or less of the size of merus in *C.tenuirostris*) (Figs 2C, D; 4G, H).

#### Family Epialtidae MacLeay, 1838

##### Subfamily Pisinae Dana, 1851

###### Nibilia

Taxon classificationAnimaliaDecapodaInachoididae

A Milne-Edwards, 1878

Nibilia A Milne-Edwards, 1878: 132, pl. 25. [Type taxon: Nibiliaerinacea A Milne-Edwards, 1878 accepted as Nibiliaantilocapra (Stimpson, 1871) by monotypy].

####### Included species.

*Nibiliaantilocapra* (Stimpson, 1871) (= *Pisapraelonga* Stimpson, 1871; = *Nibiliaerinacea* A Milne-Edwards, 1878 subjective junior synonyms); *Nibiliamachala* sp. n.

####### Emended diagnosis.

Carapace densely covered with several long and short spines and acute tubercles. Rostrum bifurcated, proximally contiguous, becoming moderately divergent distally. Preorbital angles prolonged into long spines; postorbital margin cup-shaped, with small medial spine on anterior margin protecting eyes when retracted. Fissure between antennal basal article and postorbital margin closed. Basal article of antenna elongated with two strong spines in outer margin. Exopod of third maxilliped with small process in dorsal face extending into posterolateral margin of merus; ventral margin with strong spine in distal third; ischium dorsal face with longitudinal, smooth, deep “L” shaped groove. G1 with three well-developed lobes.

###### Nibilia machala sp. n.

Taxon classificationAnimaliaDecapodaInachoididae

http://zoobank.org/D19B3071-E66E-4194-B2C4-B90859EE165F

Figure 5A, C, E

####### Holotype.

Ecuador, off Machala, near Isla Santa Clara, Southeast Pacific Biological Oceanographic Project (SEPBOP), R/V Anton Bruun, Cruise 18B, stn 771, 03°15'S; 80°50'W, 10.ix.1966, Smithsonian Oceanographic Sorting Center coll., 77–80 m, juvenile female, cl 58.4 mm, cw 35.00 mm (USNM 1462701).

####### Comparative material.

*Nibiliaantilocapra*. United States of America, North Carolina, R/V Oregon II, stn 10695, 35°22'N; 74°57'W, 26.vii.1969, HB Roberts det., 104 m, 1 ovigerous female (USNM 1256400). Louisiana, Gulf of Mexico, R/V Pelican, stn NSF–RHODOLITH–28, 27°58.925'N; 91°39.831'W, 6.v.2018, 72 m, 1 juvenile (USNM 1479292). Florida, Tortugas, southeast from n° 2 red buoy, 17.vi.1932, MJ Rathbun det., 1 male (USNM 72957). Mexico, Gulf of Mexico, Suez and Campeche, R/V Oregon, stn 406, 22°14'N; 91°26'W, 16.viii.1951, FA Chace det., 1 female (USNM 92648). Gulf of Mexico, R/V Pelican, stn 10, 4.ii.1938, W Anderson; M Lindner coll., 1 female (USNM 1236185). Southwest Gulf of Mexico, R/V Pelican, stn NFS–11–034, 9.vi.2005, DL Felder coll., 94–93 m, 1 female, DNA only (ULLZ 7365). Nicaragua, R/V Oregon, stn 6426, 12°56'N; 82°21'W, 05.ii.1967, DJ Griffin det., 190 m, 1 female juvenile (USNM 1256401). Venezuela, R/V Oregon, stn 5641, 11°38'N; 69°27'W, 01.x.1965, DJ Griffin det., 55 m, 1 male juvenile (USNM 1256402). Guyana, R/V Oregon II, stn 10513, 08°26'N; 58°11'W, 27.iv.1969, DJ Griffin & HB Roberts det., 183 m, 1 male (USNM 1256403). Brazil, Pernambuco, CEPEMAR, stn 54 L2, 150 m, 2 females (MOUFPE 15488). Bahia, Alfredo coll., 1 male (MZUSP 20258).

####### Type-locality.

Ecuador, off Machala, near Isla Santa Clara, 03°15'S; 80°50'W, 77–80 m.

####### Diagnosis.

Carapace pyriform, very spinulose, with one small spine on each side of the contiguous portion of the rostrum; one long, acute supraorbital spine; hepatic region with two long, distinct spines. Merus of P2 smooth.

####### Description.

Carapace pyriform, longer than wide, with seven long lateral spines, eight spines in medial line; covered with sparsely distributed tufts of hooked setae, mainly in rostral, branchial regions. Carapace spines: base of rostrum with five spines, two between the orbits; two protogastric; six mesogastric; six metagastric; one urogastric; two long lateral, two smaller mesial hepatic. Branchial region with small sparse tubercles and short spines: eight protobranchial, with few interspaced tubercles; mesobranchial with six long lateral, four small mesial spines, few tubercles; three marginal metabranchial with acute tubercles interspaced; seven cardiac; two intestinal spines. Branchiostegal region with row of acute, strong spines along anterior-inferior half of molt line. Gastric, branchial, cardiac, intestinal regions delimited laterally by shallow grooves.

Rostrum long, bifurcated for distal 1/3 of entire length, divergent. Supraorbital spine long, acute; orbital margin with one small spine. Postorbital margin cup-shaped completely protecting eyes when retracted, with small medial spine on anterior margin. Basal article of antenna narrow, second article long with one long anterolateral spine aligned with supraorbital spine, one smaller posterolateral spine protecting eyestalk from below, one smaller spine below orbital fissure. Antenna almost exceeding rostral length (flagellum broken in holotype). Antennal article longest; third, fourth antennal articles thick, cylindrical; visible dorsally. Antennular fossae longitudinally ovate, longer than wider; posteroventral margin with one small projection. Interantennular septum long, compressed laterally, forming ventrally-directed keel.

Epistome narrower, more depressed than antennular fossae; posterior margin crenulate, antennal gland open in epistome with one tubercle at same level, another on mouthfield border. Endostome with two prominent, obliquely longitudinal endostomial ridges, completely closed.

Buccal field sub-rectangular, longer than wide, posterior edge narrower, with crenulated anterolateral angles with one strong acute spine on anterolateral margins. Pterygostomial region sub-triangular with four acute spines on lateral margin, 3–4 sub-equal tubercles; sub-hepatic region delimited from pterygostome by distinct slope.

Third maxillipeds completely covering buccal frame. Exopod long, nearly reaching distal margin of merus; dorsal face with one small process extending into posterolateral margin of merus; ventral margin with strong spine in distal third. Ischium distinctly longer than broad, dorsal face with longitudinal, smooth, deep “L” shaped groove; crista dentata with small, rounded, irregular sized teeth. Merus slightly longer than half of ischium, anteromesial border partially covering propodus; anterior margin deeply incised, anterolateral margins slightly expanded. Palp cylindrical, slightly overreaching ischiomeral suture. Carpus, propodus, dactylus smooth; propodus, with long distomesial setae, dactylus fringed with row of long setae.

Juvenile female thoracic sternites I–IV fused, broadly triangular, smooth, dense, covered by closely adhered pubescence. Anterior half of fused sternites I–IV sloping down in ventral view. Sterno-pleonal cavity completely closed by telson. Female sternites V–VII smooth; Margin of episternites IV–VII smooth.

Juvenile female pleonal somites I–VI, telson free, slightly raised medially forming low longitudinal ridge. One small spine in first somite; somites II–VI smooth. Telson triangular. Juvenile female holotype with a sealed pleon.

Juvenile female chelipeds subequal, long; ischium unarmed; merus armed with seven strong dorsal spines, row of six laterodistal tubercles, sparse tubercles present; carpus with sparse tubercles; propodus smooth; dactylus and fixed finger distinctly shorter than palm, slender, cutting edges with subequal teeth, tip incurving down. P2 slender, cylindrical, with a distinct spine in distal margin of merus, densely covered with small setae and sparse hooked setae. Only P2 preserved in the holotype.

####### Distribution.

Only known from the type-locality in Ecuador, near Isla Santa Clara, 03°15'S; 80°50'W.

####### Etymology.

The specific epithet *machala* is a noun in apposition referring to the coastal city of Machala, Ecuador.

####### Remarks.

**Figure 5.**
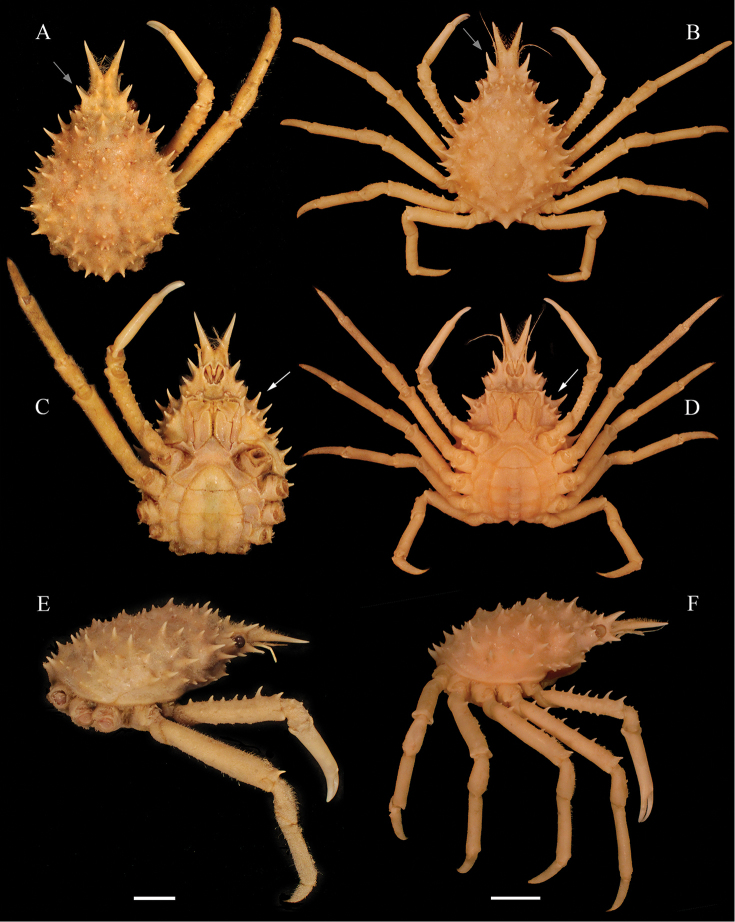
*Nibiliamachala* sp. n., female holotype, cl 58.4 mm, cw 35 mm (USNM 1462701) (**A, C, E**). *Nibiliaantilocapra*Stimpson (1871), female, cl 47.6 mm, cw 30.6 mm (USNM 1462686) (**B, D, F**). Habitus, dorsal view (**A, B**); Ventral view (**C, D**); Lateral view (**E, F**). Note the difference between the interorbital region spines (gray arrows) (**A, B**), and the hepatic spines (white arrows) (**C, D**). Scale bars: 10 mm.

*Nibiliamachala* superficially resembles *N.antilocapra* (Stimpson, 1871) in the highly spinulose appearance of the carapace, the long and semi-contiguous rostral spines, the distinct “L” shaped sulcus imprinted on the dorsal margin of the ischium of the third maxilliped, P2 with a distinct spine on the dorso-distal margin of merus, and similar shape of the female pleon (Fig. 5). *Nibiliamachala*, however, differs from *N.antilocapra* by (i) the presence of one small spine on each side of the contiguous portion of the rostrum (vs. the contiguous portion of the rostrum unarmed in *N.antilocapra*) (Fig. 5A, B, black arrow); (ii) one long, acute supraorbital spine (vs. two acute spines, one long and one shorter in *N.antilocapra*) (Fig. 5A, B); (iii) hepatic region with two long, distinct spines (vs. one long, distinct spine in the hepatic region of *N.antilocapra*) (Fig. 5C, D, white arrow); (iv) P2 merus smooth (vs. P2 armed with two rows of six or seven spines in *N.antilocapra*) (Fig. 5E, F). *Pisapraelonga* Stimpson, 1871, is apparently a juvenile stage collected at the same locality of *N.antilocapra*, thus, considered a junior subjective synonym of *N.antilocapra* (Rathbun 1925; see also figures on Milne-Edwards and Bouvier 1923: pl 11, Figs 4–7). *Nibiliaerinacea* A Milne-Edwards, 1878 is also considered a junior subjective synonym of *N.antilocapra*. The description and the figure presented by A Milne-Edwards (1878: 133, pl 25) fully agree with the description of *Pisaantilocapra* Stimpson, 1871. Both, *Pisapraelonga* and *Nibiliaerinacea* where described from western Atlantic material.

#### Family Epialtidae MacLeay, 1838

##### Subfamily Pisinae Dana, 1851

###### Solinca gen. n.

Taxon classificationAnimaliaDecapodaInachoididae

http://zoobank.org/0DD6BA19-F0F6-401F-92C7-FE271F607D2A

####### Type species.

*Solincaaulix* gen. n. et sp. n. by monotypy and original designation. Gender feminine.

####### Diagnosis.

Carapace distinctly sub-circular in outline, dorsal surface prominently vaulted, particularly swollen at branchial regions. Urogastric region compressed by metabranchial lobes into a deep furrow. Four spines along dorsal midline of carapace: mesogastric, metagastric, cardiac, and intestinal. Branchiostegal region with two rows of small acute spines along molt line. Thoracic pleurites V–VII gymnopleura. Postorbital spine long, curved beyond eyes. Eyes not retractable. Endostomial ridge with two obliquely longitudinal, very curved prominences. Sterno-pleonal cavity longer than pleon plus telson, leaving gap between distal end of telson and its anterior margin. Gonopod reaching far beyond thoracic sternal suture IV/V, rather straight proximally and medially, distinctly curved inwards sub-distally, convergent anteriorly, apical plate curved down with three distinct lobes laterally.

####### Etymology.

The genus name is an arbitrary noun formed by the combination of the Latin *Solis*, “sun” and alluding to the sub-circular carapace surrounded by spines, and *Inca* alluding to the Inca Empire. Gender: feminine.

####### Remarks.

*Solinca* is phylogenetically allied to the epialtids *Scyraacutifrons*, *Pugettianipponensis, Pugettiaquadridens* and *Chorilialongipes* (Fig. 1). However, the only morphological character shared by these taxa is a distinct sulcus in each face of the chelipeds, albeit this character is less pronounced in *Solinca*. Considering overall morphological similarities, *Solinca* is closest to *C.longipes* in the following characters: (i) presence of several spines on the carapace in both *Solinca* and *C.longipes* (vs. carapace tuberculate or with strong spines in *S.acutifrons*, *P.nipponensis*, and *P.quadridens*); (ii) supraorbital spines acute in both *Solinca* and *C.longipes* (vs. short and truncated supraorbital spines in *S.acutifrons*, *P.nipponensis*, and *P.quadridens*.); (iii) rostral spines rounded (vs. rostral spines flatted in *S.acutifrons*, *P.nipponensis* and *P.quadridens*); (iv) long and very thin legs in *Solinca* and *C.longipes* (vs. short and robust legs in *S.acutifrons*, *P.nipponensis*, and *P.quadridens*); and (v) thoracic pleurites V–VII gymnopleura in *Solinca* and *C.longipes* (vs. gymnopleura condition not present in *S.acutifrons*, *P.nipponensis*, and *P.quadridens*). See remarks of the species for differences between *S.aulix* and *Chorilialongipes*.

###### Solinca aulix sp. n.

Taxon classificationAnimaliaDecapodaInachoididae

http://zoobank.org/4A973066-37F9-4705-BBA1-BA21C057832

Figures 6A–F , 7D–G

####### Holotype.

Peru, off Paita, Piura, Southeast Pacific Biological Oceanographic Project (SEPBOP), R/V Anton Bruun, stn 627–A, 05°01' / 05°02'S; 81°25'/ 81°24'W, 03.vi.1966, Smithsonian Oceanographic Sorting Center coll., 200–311 m, male holotype, cl 37.3 mm, cw 28.2 mm (USNM 1462734).

####### Paratypes.

Peru, off Paita, Piura, Southeast Pacific Biological Oceanographic Project (SEPBOP), R/V Anton Bruun, stn 627–A, 5°01' / 05°02'S; 81°25' / 81°24'W; 3.vi.1966, Smithsonian Oceanographic Sorting Center coll., 200–311 m, 1 female (MZUSP 38891), 1 male (MZUSP 38892). Idem, 1 female, cl 39.5, cw 29.8 mm, 1 male and 1 juvenile (USNM 1462685). Southeast Pacific Biological Oceanographic Project (SEPBOP), R/V Anton Bruun, cruise 16, stn 635-A, 06°45'S; 80°93'W, 5.ix.1966, Smithsonian Oceanographic Sorting Center coll., 160 m, 1 male, cl 40.31 mm, cw 32.05 mm (USNM 1462673).

####### Material examined.

Ecuador, Gulf of Guayaquil, northwest of Tumbes, Southeast Pacific Biological Oceanographic Project (SEPBOP), R/V Anton Bruun, stn 768, 03°39'S; 80°41'W, 10.ix.1966, Smithsonian Oceanographic Sorting Center coll., 13 m, 1 juvenile female (USNM 1460378). Peru, off Paita, Piura, Southeast Pacific Biological Oceanographic Project (SEPBOP), R/V Anton Bruun, stn 627-A, 05°01' / 05°02'S; 81°25' / 81°24'W, 03.vi.1966, Smithsonian Oceanographic Sorting Center coll., 200–311 m, 6 males, 3 juveniles males, 5 females, 1 ovigerous females (USNM 1462735), off Isla Lobos de Tierra, Southeast Pacific Biological Oceanographic Project (SEPBOP), R/V Anton Bruun, stn 635A, 06°27' / 06°23'S; 80°56' / 80°55'W, 05.vi.1966, Smithsonian Oceanographic Sorting Center coll., 160 m, 4 males, 7 ovigerous female (USNM 1462736).

####### Comparative material.

*Chorilialongipes* Dana, 1851. Canada, British Columbia, Queen Charlotte Islands, Port Hardy, United States Fish Commission, R/V Albatross, stn 2862, 50°49'N; 127°36'W, 1.ix.1888, 3 males, 5 females, 2 juveniles (USNM 15497). United States of America, Alaska, vicinity of Yes Bay, Behm Canal, east end Square Island, Spacious Bay S, 48W, 19 miles, 130–193 m, 8.vii.1903, 2 males, 2 females (USNM 31637). California, Farallon Island, R/V Velero, EPA Farallon Study Expedition, stn 1, R Carney coll. det., 17 specimens (USNM 1420706). California, NE of Santa Barbara Island, Channel Islands, United States Fish Commission, R/V Albatross, stn 4416, 591–819 m, 12.iv.1904, 13 specimens (USNM 46534).

*Pugettianipponensis* Rathbun, 1932. Japan, Honshu Island, Doumiki-saki, R/V Albatross, stn 3771, 05.vi.1900, MJ Rathbun det., male holotype (USNM 48254).

*Pugettiaquadridens* (De Haan, 1839). South Korea, Dolsan Island, Sea of Japan, 1 juvenile, DNA only (ULLZ 13538). Japan, Honshu Island, Suruga Bay, Omae Zaki, R/V Albatross, stn 3730, 16.v.1900, MJ Rathbun det., 1 male, 1 female (USNM 49925).

*Scyramathiavesicularis* Rathbun, 1907. Ecuador, South of Española, Galapagos Islands, 1°50'83"S; 89°58'33"W, R/V Albatross, stn 4642, 549 m, 7.xi.1904, MJ Rathbun det., male holotype (USNM 32860).

*Scyraacutifrons*Dana 1851. United States of America, Washington, Port Orchard, Puget Sound, vi.1889, OB Johnson coll., MJ Rathbun det., 8 males, 3 females (USNM 14966). Washington, Lopez Island, Rock Point, 48N; 122W, G Paulay coll., 22.vi.2007, DNA only (UF 11955).

####### Type-locality.

Peru, off Paita, Piura, 05°01'S to 05°02'S; 81°25'W to 81°24'W, 200–311 m.

####### Diagnosis.

Same as for the genus.

####### Description.

Carapace distinctly sub-circular in outline, surface prominently inflated, particularly swollen at protogastric and branchial regions. Urogastric region compressed by metabranchial lobes into a deep furrow. Four spines - mesogastric, metagastric, cardiac, intestinal – along dorsal carapace midline. Dorsal carapace sparsely covered with long simple and hooked setae. Gastric region with two lateral spines, one protogastric, one mesogastric. One hepatic, one small sub-hepatic spines. Branchial region with four protobranchial spines; mesobranchial with four long, five shorter spines; two metabranchial, one lateral, one mesial spine above metabranchial lobe. Mesial border of branchial region with one distinct spine near the furrow, one cardiac and one intestinal spine. Branchiostegal region with two rows of spines, superior row with five strong, acute spines along most of posteroinferior half of molt line, at least five smaller spines in lower row. Gastric region delimited by shallow grooves; branchial, cardiac, intestinal regions delimited by well-marked grooves. Gastric, branchial regions with few tubercles or small spines. Pterygostomial region sub-triangular with five acute spines, few tubercles on lateral margin, smooth medially, inflated, visible in dorsal view. Thoracic pleurites V–VII gymnopleura.

Rostrum bifurcated, short, straight, more divergent in juveniles. Supraorbital spine acute, pointed forward. Postorbital spine long, curved beyond eyes. Eyes not retractable. Basal article of antenna narrow, second article long with two spines, one anterolateral, one posterolateral; one small sub-orbital spine in line with antennal gland. Antennae exceeding the rostral length, visible dorsally, flagellum short, thin; third antennal article longest; third and fourth antennal articles thick, cylindrical. Antennular fossae longitudinally ovate, longer than wide; interantennular septum long compressed laterally, forming ventrally-directed keel.

Epistome narrower than antennular fossae, anterior margin smooth, posterior margin crenulated; antennal gland open in epistome. Endostome with two obliquely prominent, longitudinal, very curved endostomial ridges. Buccal field sub-rectangular, longer than wide, narrower at posterior edge with smooth anterolateral angles.

Third maxillipeds covering buccal frame posteriorly, incompletely covering in anterior margin. Exopod long, nearly reaching distal margin of merus; ventral face with small process extending to posterolateral margin of merus. Ischium distinctly longer than broad, dorsal face smooth, deeply sculpted; crista dentata with very small, rounded teeth. Merus slightly longer than half of ischium, anteromesial border partially covering the propodus; anterior margin deeply incised, anterolateral margins slightly expanded, rounded. Palp cylindrical, slightly overreaching ischiomeral suture. Carpus, propodus and dactylus smooth; Propodus short, dactylus long and thin, with row of long setae on the distal margin. Male chelipeds equal, long, strong; merus, carpus and propodus sculpted by distinct sulcus in lateral and mesial faces; ischium smooth; merus armed with four dorsal spines, two smaller ventral spines; carpus with 3–4 blunt tubercles; propodus smooth; dactylus and fixed finger smooth, with same size as palm, cutting edges with sub-equal teeth in distal half, distinct proximal tooth in larger males; juvenile males and females fingers without gap.

P2–P5 long, slender, cylindrical, armed with distinct spine in distal margin of merus. P2 much longer than cheliped; P3–P5 progressively decreasing in length. Females with long, slender chelipeds. All legs covered with sparse, long simple setae.

Male thoracic sternite I-IV fused, broadly triangular, smooth; posterior half strongly sloping down in ventral view, forming a carina along lateral margin of telson. Sterno-pleonal cavity longer than telson, leaving gap between telson and anterior margin. Male sternites V–VII smooth; sternite VIII extending laterally beyond sterno-pleonal cavity, visible in ventral view. Margin of male episternites IV–VII smooth; female episternites IV–VII smooth, densely covered with small pubescence.

Male pleonal somites I–VI, telson free, smooth, slightly raised medially forming a low longitudinal ridge; first somite with distinct spine. Female pleonal somites I–IV, telson free; pleon markedly arched covering entire sterno-pleonal cavity; second somite with a distinct tubercle, sometimes forming a spine. Telson sub-triangular, terminating in rounded apex in males; female telson transversely oval.

First gonopod longer than thoracic sternal suture IV-V, straight proximally and medially, distinctly curved inwards sub-distally, convergent anteriorly; apical plate curved down with three well-pronounced lobes. Mesial lobe small, densely spinulate, curving toward sternal margin; distal lobe bilobed, long, tip rounded upwards; lateral lobe shorter than distal lobe, curved upward. G2 slender, straight, about 1/4 of G1 total length.

####### Distribution.

Ecuador, from Tumbes to Peru, Isla Lobos de Tierra at depths between 13 to 311 m.

####### Etymology.

The specific epithet *aulix* is the feminine Latin noun for “furrow” or “sulcus”, and alludes to the furrow in the intestinal region formed by the junction of the highly inflated branchial regions.

####### Remarks.

**Figure 6.**
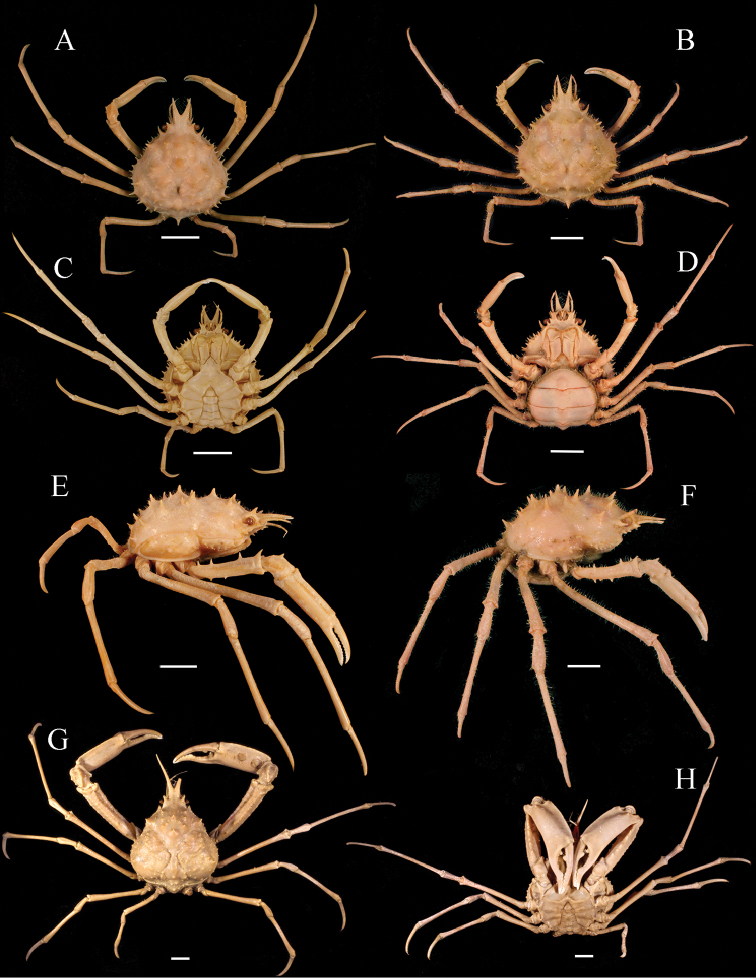
*Solincaaulix* gen. n. et sp. n. (**A–F**), male holotype, cl 37.30 mm, cw 28.2 mm, (USNM 1462734) (**A, C, E**); Female paratype, cl 39.5 mm, cw 29.8 mm (USNM 1462685) (**B, D, F**). *Chorilialongipes* Dana, 1851, male, cl 56.5 mm, cw 48.1 mm (USNM 46534) (**G, H**). Habitus, dorsal view (**A, B, G**); Ventral view (**C, D, H**); Lateral view (**E, F**). Scale bars: 10 mm.

**Figure 7.**
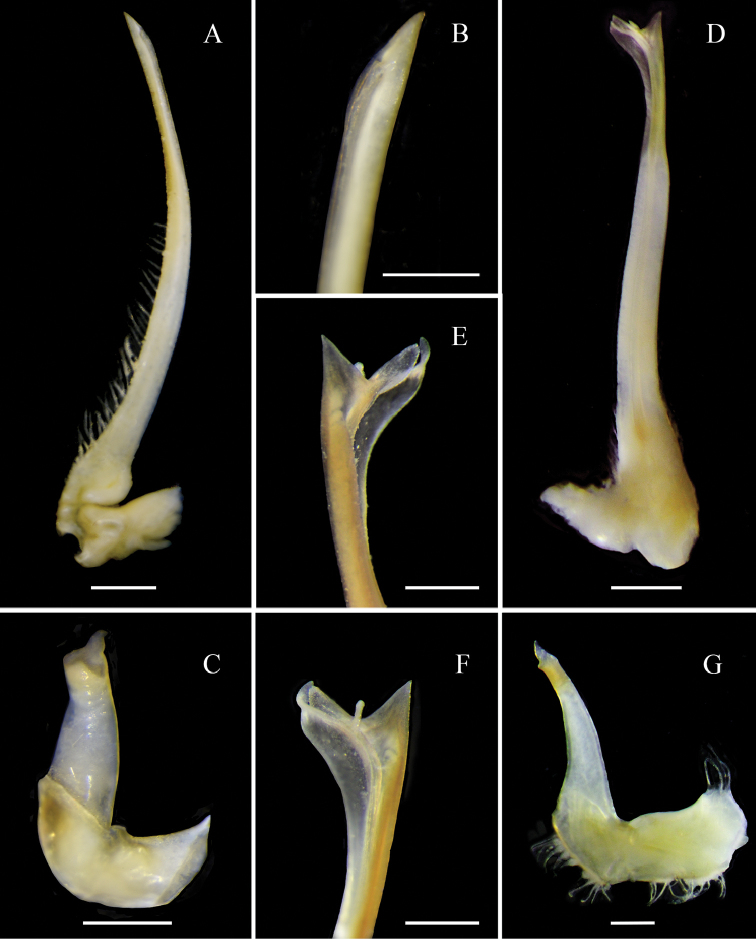
*Collodesanartius* sp. n., male paratype (USNM 142821) (**A–C**); First gonopod (G1), right side, sternal view (**A**), pleonal view (**B**); Second gonopod (G2), right side (**C**). *Solincaaulix* gen. n. et sp. n., paratype male (USNM 1462673) (**D–G**); First gonopod (G1), left side, pleonal view (**D, F**), sternal view (**E**); Second gonopod (G2), left side (**G**). Scale bars: 1 mm (**A, D**); 0.5 mm (**B, C, E–G**).

*Solincaaulix* can be distinguished from *Chorilialongipes* by a unique set of characters, which include: (i) rostral spines of *Solincaaulix* shorter than *C.longipes* (Fig. 6A, B, G); (ii) postorbital spines long, curved beyond eyes in *Solinca* (vs. truncated postorbital process curved medially in *C.longipes*) (Fig. 6A, B, G); (iii) protogastric and branchial regions distinctly swollen in *Solincaaulix* (vs. protogastric and branchial regions weakly swollen in *C.longipes*) (Fig. 6A, B, G); (iv) urogastric region compressed by metabranchial regions forming a furrow in *Solincaaulix* (vs. urogastric region not compressed and with some tubercles in *C.longipes*) (Fig. 6A, B, G); (v) anterolateral border of the merus of the third maxilliped rounded in *Solincaaulix* (vs. anterolateral border of the merus of the third maxilliped pointed in *C.longipes*) (Fig. 6C, D, H); (vi) pterygostomial region inflated, visible in dorsal view in *Solincaaulix* (vs. pterygostomial region not inflated and not visible in dorsal view in *C.longipes*) (Fig. 6C, D, H); (vii) third and fourth antennal articles short and cylindrical in *Solincaaulix* (vs. third and fourth antennal articles long and flattened in *C.longipes*); and (viii) G1 slightly overreaching the thoracic sternal suture IV-V in *Solincaaulix* (Fig. 7D–G) (vs. G1 distinctly overreaching the thoracic sternal suture IV-V in *C.longipes*).

## Supplementary Material

XML Treatment for Collodes

XML Treatment for Collodes anartius

XML Treatment for Nibilia

XML Treatment for Nibilia machala

XML Treatment for Solinca

XML Treatment for Solinca aulix

## Supplementary materials

Supplementary material 1 Taxa included in the molecular phylogenetic analyses to place the newly described taxa within the context of the superfamily Majoidea Data type: phylogenetic data This is an open access article distributed under the terms of the Creative Commons Attribution License (CC BY 4.0), which permits unrestricted use, distribution, and reproduction in any medium, provided the original author and source are credited.Jessica Colavite, Amanda Windsor, William Santana
