# Supplementary material for: Three new species and a new genus of majoid crabs from the eastern Pacific (Decapoda, Brachyura)
Source: Zookeys. 2019 Feb 18;(825):1–24. doi: 10.3897/zookeys.825.32271 (PMC6389645; doi:10.3897/zookeys.825.32271)
Supplement: Supplementary material 1 [file zookeys-825-001-s001.docx]

**Supplementary Table 1.** Taxa included in the molecular phylogenetic analyses to place the newly described taxa within the context of the superfamily Majoidea. Catalog number abbreviations NTOU, National Taiwan Ocean University; CCDB, Crustacean Collection of the Department of Biology, FFCLRP, University of São Paulo; CIB, Pacific Biological Station, Fisheries and Oceans CA; MNHN, Muséum National d’Historie Naturelle, Paris; MZUSP, Museum of Zoology of University of São Paulo; UF, Florida Museum of Natural History; ULLZ, University of Louisiana at Lafayette Zoological Collection; USNM, United States National Museum, Smithsonian Institution; ZRC, Zoological Research Collection Lee Kong Chian Natural History Museum, Singapore. Atl. = Atlantic Ocean; Car. = Caribbean Sea; GMx = Gulf of Mexico; IN = Indian Ocean; Med. = Mediterranean Sea; Pac. = Pacific Ocean. Water body names preceded by N, S, E, or W to indicate northern, southern, eastern or western part of basin, respectively; — = no sequence.

| **Taxon Name** | **Basin /Country** | **Catalog No.** | **GenBank Accession Nos.** | | | | |
| --- | --- | --- | --- | --- | --- | --- | --- |
|  |  |  | **12S** | **16S** | **COI** | **18S** | **H3** |
| **New Taxa** | | | | | | | |
| *Collodes* *anartius* sp. n. (holotype) | E Pac., PE | USNM 1462817 | MK309543 | MK309608 | MK281271 | — | — |
| *Collodes anartius* sp. n. (paratype) | E Pac., PE | USNM 1462747 | MK309542 | MK309607 | — | — | — |
| *Collodes anartius* sp. n. (paratype) | E Pac., PE | USNM 1462821 | MK309544 | MK309609 | — | — | — |
| *Collodes anartius* sp. n. (paratype) | E Pac., PE | USNM 1462676 | MK309541 | — | MK281270 | — | — |
| *Nibilia machala* sp. n. (holotype) | E Pac., EC | USNM 1462701 | MK309567 | MK309606 | MK281281 | — | — |
| *Solinca aulix* sp. n. (holotype) | E Pac., EC | USNM 1462734 | MK309532 | MK309572 | MK281261 | — | — |
| *Solinca aulix* sp. n (paratype) | E Pac., PE | USNM 1462673 | MK309562 | — | — | — | — |
| **Majoidea** | | | | | | | |
| *Acanthonyx lunulatus* | E Atl., CV | ULLZ 11713 | — | KF452983 | KF452903 | — | KF453159 |
| *Acanthonyx petiverii* | W GMx | ULLZ 8480 | MK309553 | MK309588 | MK281285 | — | — |
| *Aepinus septemspinosus* | SE GMx | ULLZ 6602 | MK309545 | MK309580 | MK281262 | — | — |
| *Aepinus septemspinosus* | N GMx | ULLZ 6676 | KF453104 | KF452998 | KF452918 | — | KF453166 |
| *Chorillia longipes* | N Pac., CA | USNM 15497 | MK309564 | MK309604 | — | — | — |
| *Chorinus heros* | Car. BZ | ULLZ 11199 | KF453081 | KF452977 | KF452900 | KF453154 | — |
| *Collodes gibbosus* | E Pac., CR | ULLZ 8229 | KF453122 | KF453014 | KF452933 | — | — |
| *Collodes granosus* | E Pac., PA | ULLZ 9760 | MK309535 | MK309590 | MK28126 | MK285650 | MK281295 |
| *Collodes inermis* | SW Atl., BR | CCDB 0112 | — | MF490172 | MF490078 | — | — |
| *Collodes leptocheles* | GMx | ULLZ 6508 | MK309534 | MK309579 | MK281263 | — | — |
| *Collodes obesus* | N GMx | ULLZ 10977 | MK309538 | MK309592 | MK281268 | — | — |
| *Collodes robustus* | E GMx | ULLZ 8349 | MK309539 | MK309587 | MK281269 | — | — |
| *Collodes tenuirostris* | E Pac., CR | ULLZ 8235 | MK309537 | MK309585 | MK281265 | MK285649 | — |
| *Collodes tenuirostris* | E Pac., PA | USNM 1479280 | MK309536 | MK309610 | MK281267 | — | — |
| *Collodes tenuirostris* | E Pac., PE | USNM 1479343 | MK309571 | MK309573 | MK281266 | — | — |
| *Collodes tenuirostris* | E Pac., EC | USNM 1155058 | MK309540 | MK309605 | — | — | — |
| *Epialtoides kingsleyi* | Car. PA | ULLZ 12252 | MK309549 | MK309597 | — | — | — |
| *Epialtus bituberculatus* | W Atl., US | ULLZ 15314 | MK309550 | MK309601 | MK281272 | — | — |
| *Epialtus bituberculatus* | Car. PA | ULLZ 10757 | KF453080 | KF452976 | KF452898 | KF453153 | — |
| *Herbstia condyliata* | E Atl., PT | Her_c_131 | — | EU682790 | EU682845 | — | — |
| *Inachoides laevis* | E Pac., US | UF 15195 | MK309533 | MK309578 | — | — | MK281296 |
| *Inachoides laevis* | E Pac., PA | ULLZ 9156 | KF453134 | KF453025 | KF452945 | KF453188 | — |
| *Inachus aguiarii* | Medit. | ULLZ 11667 | MK309546 | MK309595 | — | MK285641 | — |
| *Inachus communissimus* | Medit. | ULLZ 11656 | MK309547 | MK309593 | — | MK285643 | — |
| *Inachus* *dorsettensis* | Medit. | ULLZ 11658 | MK309548 | MK309594 | — | MK285642 | — |
| *Leptopisa setirostris* | SW GMx | ULLZ 6994 | MK309569 | MK309582 | MK281278 | MK285645 | MK281294 |
| *Leptopisa setirostris* | SW GMx | ULLZ 6798 | KF453107 | KF453000 | KF452921 | KF453170 | KF453049 |
| *Leurocyclus tuberculosus* | SW Atl., BR | CCDB 1768 | — | MF490185 | — | — | — |
| *Libinia dubia* | W Atl., US | ULLZ 12205 | MK309570 | MK309596 | MK281279 | — | — |
| *Libinia emarginata* | N GMx | ULLZ 10344 | KF453078 | KF452974 | KF452896 | KF453151 | — |
| *Libinia erinacea* | W Atl., US | ULLZ 7747 | KF453119 | KF453011 | KF452932 | — | — |
| *Libinia ferreirae* | SW Atl., BR | CCDB 956 | — | MF490186 | MF490093 | — | — |
| *Macrocoeloma camptocerum* | SW GMx | ULLZ 6889 | KF453110 | KF453003 | KF452924 | KF453173 | KF453048 |
| *Macrocoeloma trispinosum* | Car. GP | MNHN-IU-2013-4312 | MK309561 | MK309574 | MK281284 | — | — |
| *Macrocoeloma trispinosum* | W Atl., US | ULLZ 4571 | KF453097 | KF453042 | KF452911 | KF453162 | — |
| *Macropodia parva* | E Atl., ES | ULLZ 11426 | KF453083 | KF452979 | KF452901 | KF453156 | — |
| *Maja crispata* | Adriatic Sea | ULLZ 11671 | KF453085 | EU000852 | — | KF453145 | — |
| *Maja brachydactyla* | E Atl., | ULLZ 11425 | KF453082 | KF452978 | MK281275 | KF453155 | — |
| *Minyorhyncha crassa* | NW GMx | ULLZ 15552 | MK309556 | MK309602 | MK281287 | — | — |
| *Minyorhyncha crassa* | N GMx | ULLZ 7774 | MK309555 | MK309584 | MK281286 | — | — |
| *Mithraculus cinctimanus* | Car. PA | ULLZ 12012 | KF453089 | KF452986 | KF452905 | — | — |
| *Mithraculus forceps* | N GMx | ULLZ 4893 | KF453098 | — | KF452912 | — | — |
| *Mithraculus sculptus* | W Atl., | ULLZ 8774 | GU144526 | GU144539 | GU144555 | — | — |
| *Mithrax pleuracanthus* | W Atl., US | ULLZ 5694 | GU144537 | GU144544 | GU144560 | — | — |
| *Mithrax tortugae* | Car. PA | ULLZ 6980 | GU144527 | GU144542 | GU144562 | — | — |
| *Mocosoa crebripunctata* | SW GMx | ULLZ 6821 | KF453108 | KF453001 | KF452922 | KF453171 | — |
| *Nibilia antilocapra* | SW GMx | ULLZ 7365 | MK309566 | MK309583 | MK281282 | MK285646 | — |
| *Nibilia antilocapra* | N GMX | USNM 1479292 | — | MK309576 | MK281283 | — | — |
| *Notolopas brasiliensis* | SW Atl., BR | CCDB 0097 | — | MF490198 | MF490104 | — | — |
| *Notolopas lamellatus* | E Pac., CR | ULLZ 8337 | MK309560 | MK309586 | MK281280 | MK285648 | — |
| *Pelia mutica* | W GMx | ULLZ 6874 | MK309551 | MK309581 | MK281276 | MK285644 | — |
| *Pelia tumida* | E Pac., PA | ULLZ 10015 | MK309552 | MK309591 | MK281277 | — | — |
| *Picroceroides tubularis* | GMx | ULLZ 6830 | KF766068 | KF766069 | KF766071 | KF766070 | — |
| *Pisa hirticornis* | — | UB2011_PH | — | KC866332 | KC866323 | — | — |
| *Pisa tetraodon* | E Atl., ES | ULLZ 11427 | KF453084 | KF452980 | KF452902 | KF453157 | — |
| *Pisoides edwardsii* | SE Pac., CL | ULLZ 9144 | MK309568 | MK309589 | MK281273 | MK285647 | — |
| *Pugettia nipponensis* | W Pac., TW | NTOU B00009 | KJ132484 | KJ132626 | — | — | KJ133183 |
| *Pugettia quadridens* | NW Pac., KR | ULLZ 13538 | MK309565 | MK309599 | MK281292 | — | — |
| *Pugettia sp.* |  | NTOU B00089 | KJ132419 | KJ132558 | — | — | KJ133115 |
| *Rochinia gracilipes* | SW Atl., BR | CCDB 4166 | — | MF490213 | MF490116 | — | — |
| *Rochinia gracilipes* | SW Atl., BR | MZUSP 14213 | MK309554 | MK309575 | — | — | — |
| *Rochinia hystrix* | S Car., CW | ULLZ 15951 | MK309558 | MK309603 | MK281289 | — | — |
| *Rochinia tanneri* | N GMx | ULLZ 12719 | MK309559 | MK309598 | MK281290 | — | — |
| *Scyra acutifrons* | NE Pac., CA | CIB 336 | — | — | KX039788 | — | — |
| *Scyra acutifrons* | NE Pac., CA | CIB 270 | — | — | KX039789 | — | — |
| *Scyra acutifrons* | NE Pac., US | UF 11955 | MK309563 | MK309577 | MK281291 | — | MK281293 |
| *Scyramathia umbonata* | N GMx | ULLZ 13797 | MK309557 | MK309600 | MK281288 | — | — |
| *Schizophroida hilensis* | Pac., US | ULLZ 10436 | KF453079 | KF452975 | KF452897 | KF453152 | KF453056 |
| *Schizophrys aspera* | W IN., RE | UF 12860 | KF453071 | KF452968 | KF452891 | KF453147 | KF453060 |
| *Stenocionops furcatus* | W Atl., US | ULLZ 8410 | KF453125 | KF453017 | KF452936 | KF453179 | — |
| *Stenocionops ovatus* | E Pac., PA | ULLZ 9747 | KF453142 | KF453032 | KF452956 | KF453190 | KF453059 |
| **Outgroup** | | | | | | | |
| *Ethusa lata* | E Pac., PA | ULLZ 12098 | KF453090 | KF452987 | — | KF453160 | — |
| *Ethusa panamensis* | E Pac., PA | ULLZ 8226 | KF453121 | KF453013 | — | KF453178 | — |
| *Ethusa sexdentata* | W Pac., PH | ZRC2018.1066 | EU636969 | EU636953 | — | — | — |
| *Ethusa* sp. | — | KC3088 | — | EU920925 | — | EU920966 | EU921061 |
